# Personalized prognostic prediction tool for high-grade neuroendocrine cervical cancer: a SEER database analysis and single-center validation

**DOI:** 10.1007/s00432-023-05414-6

**Published:** 2023-10-18

**Authors:** Xiaoyue Chen, Wenpei Shi, Chao Wang, Haiyan Zhu

**Affiliations:** 1 Gynecological Department, Shanghai First Maternity and Infant Health Hospital, No. 2699 West Gao Ke Road, Pudong, Shanghai, China; 2 Clinical Research Unit, Shanghai First Maternity and Infant Health Hospital, Shanghai, China

**Keywords:** Cervical high-grade neuroendocrine carcinoma (CHGNEC), Overall survival, Prognosis, Nomogram

## Abstract

**Purpose:**

Cervical high-grade neuroendocrine carcinoma (CHGNEC) is a rare but highly aggressive cancer. The purpose of this study is to develop a prognostic nomogram that can accurately predict the outcomes for CHGNEC patients.

**Methods:**

We analyzed clinical data from the Surveillance, Epidemiology, and End Results (SEER) database of CHGNEC patients, including small-cell neuroendocrine carcinoma (SCNEC) and large-cell neuroendocrine carcinoma (LCNEC). We investigated patient characteristics and prognosis, and developed a prognostic nomogram model for cancer-specific survival in CHGNEC patients. External validation was conducted using real clinical cases from our hospital.

**Results:**

Our study included 306 patients from SEER database, with a mean age of 49.9 ± 15.5 years. Most of the patients had SCNEC (86.9%). Among them, 170 died from the disease, while 136 either survived or died from other causes. Our final predictive model identified age at diagnosis, stage 1 status, stage 4 status, T1, N0, and surgery of the primary site as independent prognostic factors for CHGNEC. We validated our model using a group of 16 CHGNEC patients who underwent surgery at our center. The external validation showed that the prognostic nomogram had excellent discriminative ability, with an area under the receiver operating characteristic curve (AUC) of 0.76 (95% CI 0.49–1.00) for the prediction of 3-year cancer-specific survival (CSS) and an AUC of 0.85 (95% CI 0.62–1.00) for the prediction of 5-years CSS. The random survival forest model achieved an AUC of 0.80 (95% CI 0.56–1.00) for 3-years CSS and 0.91 (95% CI 0.72–1.00) for 5-years CSS, indicating its adequacy in predicting outcomes for CHGNEC patients.

**Conclusion:**

Our study provides an excellent nomogram for predicting the prognosis of CHGNEC patients. The prognostic nomogram can be a useful tool for clinicians in identifying high-risk patients and making personalized treatment decisions.

**Supplementary Information:**

The online version contains supplementary material available at 10.1007/s00432-023-05414-6.

## Introduction

Cervical neuroendocrine neoplasms (CNEN) are rare and aggressive cancers that account for only 1.4% of all cervical cancer cases (Tempfer et al. 2018). These neoplasms are subdivided into typical carcinoid, atypical carcinoid, small-cell neuroendocrine carcinoma (SCNEC), and large-cell neuroendocrine carcinoma (LCNEC) (Guadagno et al. 2016). SCNEC and LCNEC are high-grade neuroendocrine carcinomas (HGNEC) and are associated with poor outcomes, even when diagnosed at an early stage. Cervix is the most common primary site in the female genital tract of HGNEC. The 5-years survival of CHGNEC was reported at 36.8% in stage I–IIA and 8.9% in IIB–IV (Cohen et al. 2010). Its prognosis is inferior to that of squamous cell carcinoma, adenocarcinoma and adenosquamous carcinoma (Margolis et al. 2016). Patients with HGNEC are more likely to experience lymphatic and hematogenous spread, recurrence, and distant metastases due to the aggressive biological behavior of the disease (Gadducci et al. 2017). Despite the rarity of CNEN, stage (Bermudez et al. 2001; Boruta et al. 2001), tumor size (Chang et al. 1998; Yin et al. 2014), lymph node status (Sukpan et al. 2011), depth of invasion (Sukpan et al. 2011), LVSI (Sukpan et al. 2011), and margin status (Chan et al. 2003) have been identified as relevant prognostic variables. However, the prognostic characteristics of patients with CHGNEC remain controversial (Gadducci et al. 2017), and there is a lack of data regarding the biology, clinical behavior, and management of such aggressive tumors. The prognosis models developed for squamous cell carcinoma and adenocarcinoma are not applicable to CHGNEC. Furthermore, studies have primarily focused on SCNEC, and corresponding data on LCNEC are even scarcer. Therefore, the development of a new prognostic model to predict cancer-specific survival (CSS) for CHGNEC is both challenging and crucial. In this study, we aimed to construct a new prognostic model based on the Surveillance, Epidemiology, and End Results (SEER) database and validated it using clinical data from our hospital.

## Methods

### Data source and inclusion criteria

This study utilized data from the Surveillance, Epidemiology, and End Results (SEER) cancer registry database as the training dataset in accordance with the SEER data use agreement. The SEER*Stat software program (version 8.3.4) was used to extract data. The pathological diagnosis was based on the primary site following the International Classification of Diseases for Oncology, third edition (ICD-O-3). Our study included histology codes 8013 (large cell neuroendocrine carcinoma), 8041 (small cell neuroendocrine carcinoma), 8240 (neuroendocrine neoplasms), and 8246 (neuroendocrine carcinoma). We limited our study to patients with high-grade neuroendocrine tumors (small cell or large cell carcinoma) and included data for postoperative lymph node status and staging from 2004 to 2015. We excluded diagnostic surgeries and included only therapeutic excisions. We utilized the 7th American Joint Committee on Cancer (AJCC) staging system in this study.

### Patient data and exclusion criteria

Sixteen patients were retrospectively studied as the validation dataset. The inclusion criteria were: (1) diagnosed with high-grade neuroendocrine tumors (small cell or large cell carcinoma) at our hospital; (2) received initial treatment between March 2007 and January 2017; and (3) diagnosed by two different pathologists according to the WHO classification of 2010. Exclusion criteria included: (1) incomplete survival data description; (2) incomplete description of metastatic status; or (3) presence of multiple primary tumors. The ethics committee of the Shanghai First Maternity and Infant Hospital, Tongji University School of Medicine, approved this retrospective study.

Demographic and clinical information, including age, grade, FIGO stage, and treatment strategies were extracted. Duration of follow-up and vital status, including the cause of death, were also included. The deadline for follow-up was December 31, 2020. Censored observations were recorded for patients alive at the last follow-up date. Survival time was defined as the duration from diagnosis to death, last contact, or December 31, 2020.

### Predictor selection, model development, and validation

Cox proportional hazards risk regression was used to identify independent prognostic predictors. The least absolute shrinkage and selection operator (LASSO) regression analysis was used to identify potential risk factors for cancer-specific death (CSD) from the training dataset. LASSO regression analysis, through cross-validation, was used to penalize the absolute value of regression coefficients, prevent overfitting of variables from the training dataset, and only retain the most effective predictors in the model. We identified six variables with a non-zero coefficient value and corresponding lambda value and likelihood of deviance, which were then ascertained into the final model.

The prediction models were developed using Cox proportional hazards risk regression analysis and random survival forest (RSF) analysis. A nomogram was constructed and validated based on Cox regression analysis to visualize and quantify the effect of each selected variable on the estimated 3- and 5-years cancer-specific survival (CSS) probability. Internal validation was performed using a bootstrap resampling method, with replacement from the training dataset, and fitting the Cox regression model and random survival forest (RSF) model in 1000 bootstrapping replicates. Receiver operating characteristic curves (ROC) and calibration curves were depicted separately for 3- and 5-years CSS. Decision-curve analysis (DCA) was used to determine the clinical net benefit associated with established predictive models. Discrimination of predictive models was quantified with the area under the curve (AUC). The dataset from our hospital (*n* = 16) was used for external validation, and the performance of the model was further estimated using the AUC.

### Statistical analysis

Continuous variables were described as mean ± standard deviation (SD) and median with interquartile range (IQR) values, while categorical variables were displayed with numbers and percentages per group. The Chi-squared, Fisher exact and Wilcoxon rank-sum tests were used to compare frequency distribution among categorical and numerical variables, respectively.

All statistical analyses were performed using R version 4.0.3 (http://www.r-project.org), with* p* < 0.05 considered statistically significant for all analyses.

## Results

### Epidemiological characteristics

This study analyzed 306 patients diagnosed with CHGNEC from the SEER database, with small cell neuroendocrine carcinoma being the most common subtype, accounting for 86.9% of cases. The mean age at diagnosis was 49.9 ± 15.5 years, and most patients were white (76.5%) and had insurance (75.5%). Lymph node metastasis was present in 45.4% of patients at diagnosis, while 36.6% had distant metastasis. Stage IV was the most common stage at presentation, accounting for 37.9% of cases. Primary treatment included cancer-directed surgery in 37.6% of patients, radiation therapy in 61.1% of patients, and chemotherapy in 77.5% of patients.

A follow-up study was conducted on the 306 patients, with 170 patients dying of CHGNEC and 136 either surviving or dying of other diseases. A comparison between the basic demographics and characteristics of patients who died of CHGNEC and those who survived or died of other diseases revealed significant differences in age at diagnosis, number of in situ/malignant tumors, insurance status, chemotherapy recode, stage, T, N, M, and whether the patient underwent surgery (*p* < 0.05). More information is provided in Table 1.

**Table 1:** Demographic and clinical characteristics of patients with CHGNEC from the SEER database

	Total (*N* = 306)	Survive/died of other diseases (*n* = 136)	Died of CHGNEC (*n* = 170)	*p* value
Histology type				0.924
SCSEC	266 (86.9%)	119 (87.5%)	147 (86.5%)	
LCNEC	40 (13.1%)	17 (12.5%)	23 (13.5%)	
Race				0.396
Black	34 (11.1%)	13 (9.6%)	21 (12.4%)	
White	234 (76.5%)	109 (80.1%)	125 (73.5%)	
Other	38 (12.4%)	14 (10.3%)	24 (14.1%)	
Age at diagnosis, years				
Mean (SD)	49.9 (15.5)	47.4 (15.6)	51.9 (15.1)	
Median [IQR]	49 [37.3, 60]	45 [35, 59]	52 [40, 61]	< 0.001
Grade				0.911
G2	3 (1.0%)	1 (0.7%)	2 (1.2%)	
G3	118 (38.6%)	55 (40.4%)	63 (37.1%)	
G4	55 (18.0%)	25 (18.4%)	30 (17.6%)	
Unknown	130 (42.5%)	55 (40.4%)	75 (44.1%)	
Marital status at diagnosis				0.630
Married	130 (42.5%)	55 (40.4%)	75 (44.1%)	
Widowed	35 (11.4%)	14 (10.3%)	21 (12.4%)	
Divorce	36 (11.8%)	15 (11.0%)	21 (12.4%)	
Single	105 (34.3%)	52 (38.2%)	53 (31.2%)	
Insurance				0.036
No	75 (24.5%)	25 (18.4%)	50 (29.4%)	
Yes	231 (75.5%)	111 (81.6%)	120 (70.6%)	
Total number of in situ/malignant tumors for patient				
Mean (SD)	1.10 (0.374)	1.21 (0.518)	1.02 (0.152)	
Median [IQR]	1 [1, 1]	1[1, 1]	1 [1, 1]	< 0.001
Chemotherapy				0.025
No/unknown	69 (22.5%)	22 (16.2%)	47 (27.6%)	
Yes	237 (77.5%)	114 (83.8%)	123 (72.4%)	
Radiation				0.132
No	119 (38.9%)	46 (33.8%)	73 (42.9%)	
Yes	187 (61.1%)	90 (66.2%)	97 (57.1%)	
Stage				< 0.001
I	57 (18.6%)	38 (27.9%)	19 (11.2%)	
II	12 (3.9%)	8 (5.9%)	4 (2.4%)	
III	75 (24.5%)	26 (19.1%)	49 (28.8%)	
IV	116 (37.9%)	32 (23.5%)	84 (49.4%)	
Unknown	46 (15.0%)	32 (23.5%)	14 (8.2%)	
T				< 0.001
T1	95 (31.0%)	52 (38.2%)	43 (25.3%)	
T2	60 (19.6%)	22 (16.2%)	38 (22.4%)	
T3	73 (23.9%)	21 (15.4%)	52 (30.6%)	
T4	14 (4.6%)	3 (2.2%)	11 (6.5%)	
TX	64 (20.9%)	38 (27.9%)	26 (15.3%)	
N				0.001
N0	104 (34.0%)	56 (41.2%)	48 (28.2%)	
N1	139 (45.4%)	46 (33.8%)	93 (54.7%)	
NX	63 (20.6%)	34 (25.0%)	29 (17.1%)	
M				< 0.001
M0	149 (48.7%)	71 (52.2%)	78 (45.9%)	
M1	112 (36.6%)	32 (23.5%)	80 (47.1%)	
MX	45 (14.7%)	33 (24.3%)	12 (7.1%)	
Cancer-directed surgery				0.014
No	191 (62.4%)	74 (54.4%)	117 (68.8%)	
Yes	115 (37.6%)	62 (45.6%)	53 (31.2%)	

Normality test for age were assessed by Shapiro–Wilk test. Comparison between groups were made using Wilcoxon rank-sum test, Chi-squared test, and Fisher exact test as appropriate

*CHGNEC* Cervical high-grade neuroendocrine carcinoma, *SD* standard deviation, *IQR* Inter quartile range

### Risk factors for cancer-specific death

To identify independent prognostic factors for CHGNEC, univariate and multivariate Cox regression analyses were performed, and the results are presented in Table 2. Univariate analysis showed that older age at diagnosis (HR = 1.03; 95% CI 1.02, 1.03), advanced tumor stages (stage III/IV vs. stage I; HR = 2.69; 95% CI 1.59, 4.58/HR = 5.03; 95% CI 3.04, 8.32), higher T stage (T4 vs. T1, HR = 5.53; 95% CI 2.83, 10.80), lymph node metastasis (HR = 2.03; 95% CI 1.43, 2.88), and distant organ metastasis (HR = 2.64; 95% CI 1.92, 3.63) were associated with increased risk of cancer-specific death. Conversely, surgery (HR = 0.45; 95% CI 0.33, 0.63), chemotherapy (HR = 0.39; 95% CI: 0.28, 0.54), and radiation therapy (HR = 0.63; 95% CI 0.47, 0.86) were identified as protective factors for CHGNEC-specific death. Subsequently, all candidate variables were included in multivariate Cox regression analysis. As shown in Table 2, eight out of 13 variables were independently associated with CHGNEC-specific survival (all *p* < 0.05), except for insurance, race, radiation, stage, N, and M. To avoid overfitting and simplify the model, LASSO regression analysis was employed to penalize the absolute value of the coefficients (Fig. S1). Based on the LASSO analysis, six variables including age at diagnosis, stage 1 status, stage 4 status, T1 status, N0 status, and surgery of the primary site were included in the final predictive model.

**Table 2:** Cox hazards regression analysis of cancer-specific survival in the development dataset with CHGNEC

Characteristics	Univariable Model		Multivariable model	
	HR (95% CI)	*p* value	aHR (95% CI)	*p* value
Histology type				
SCNEC	Ref		Ref	
LCNEC	1.20 (0.78–1.87)	0.410	1.78 (1.08–2.94)	0.023
Race				
Black	Ref		Ref	
White	0.67 (0.42–1.06)	0.087	0.80 (0.48–1.36)	0.417
Other	0.99 (0.55–1.78)	0.976	1.09 (0.57–2.08)	0.804
Age at diagnosis	1.03 (1.02–1.04)	< 0.001	1.02 (1.01–1.04)	0.002
Grade				
G2	Ref		Ref	
G3	0.87 (0.21–3.56)	0.846	0.28 (0.06–1.27)	0.098
G4	0.78 (0.19–3.26)	0.732	0.20 (0.04–0.96)	0.044
Unknown	1.02 (0.25–4.16)	0.977	0.26 (0.05–1.19)	0.082
Marital status at diagnosis				
Married	Ref		Ref	
Widowed	1.58 (0.97–2.57)	0.065	0.66 (0.36–1.19)	0.167
Divorce	1.03 (0.64–1.68)	0.894	0.95 (0.56–1.61)	0.857
Single	0.79 (0.56–1.13)	0.196	0.60 (0.41–0.89)	0.010
Insurance				
No	Ref		Ref	
Yes	0.90 (0.65–1.26)	0.537	1.00 (0.69–1.44)	0.980
Total number of in situ/malignant tumors for patient	0.28 (0.11–0.72)	0.008	0.17 (0.06–0.45)	< 0.001
Chemotherapy	Ref			
No/unknown	0.39 (0.28–0.54)	< 0.001	ref	
Yes			0.35 (0.23–0.53)	< 0.001
Radiation	Ref			
No	0.63 (0.47–0.86)	0.003	Ref	
Yes			0.75 (0.51–1.08)	0.123
Stage				
I	Ref		Ref	
II	1.14 (0.39–3.35)	0.816	0.75 (0.21–2.71)	0.658
III	2.69 (1.59–4.58)	< 0.001	2.01 (0.89–4.54)	0.093
IV	5.03 (3.04–8.32)	< 0.001	2.25 (0.48–10.66)	0.305
Unknown	2.85 (1.42–5.73)	0.003	2.69 (0.63–11.38)	0.179
T				
T1	Ref		Ref	
T2	1.89 (1.22–2.93)	0.004	0.93 (0.52–1.66)	0.796
T3	3.16 (2.11–4.76)	< 0.001	1.11 (0.65–1.9)	0.700
T4	5.53 (2.83–10.8)	< 0.001	2.80 (1.06–7.39)	0.038
TX	2.25 (1.37–3.69)	0.001	0.79 (0.39–1.58)	0.503
N				
N0	Ref		Ref	
N1	2.03 (1.43–2.88)	< 0.001	1.52 (0.93–2.48)	0.091
NX	2.5 (1.56–4)	< 0.001	1.22 (0.62–2.41)	0.560
M				
M0	Ref		Ref	
M1	2.64 (1.92–3.63)	< 0.001	1.49 (0.39–5.66)	0.557
MX	1.43 (0.77–2.65)	0.259	0.62 (0.18–2.12)	0.451
Cancer-directed surgery				
No	Ref		Ref	
Yes	0.45 (0.33–0.63)	< 0.001	0.53 (0.35–0.81)	0.003

*CHGNEC* Cervical high-grade neuroendocrine carcinoma, *HR* hazard ratio, *aHR* adjusted hazard ratio

### Prediction model construction and internal validation

**Fig. 1:**
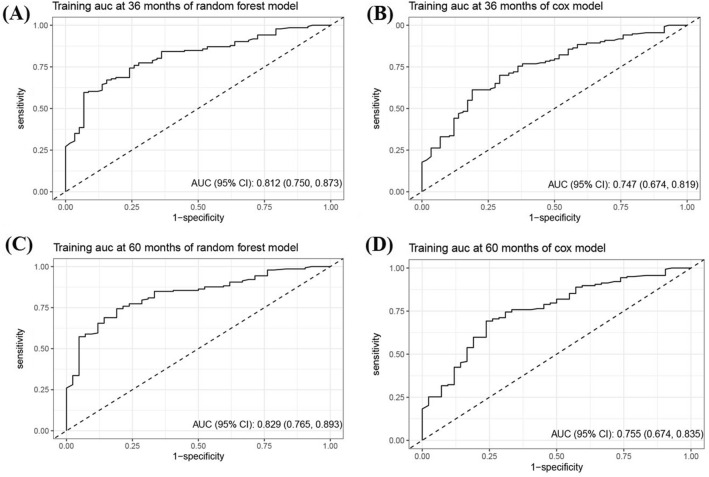
The ROC curve of predictive models for 3- and 5-year CSS in patients with CHGNEC in the development dataset. *ROC* receiver operating characteristic curves, *CSS* cancer-specific survival, *CHGNEC* cervical high-grade neuroendocrine carcinoma, *AUC* area under curve

**Fig. 2:**
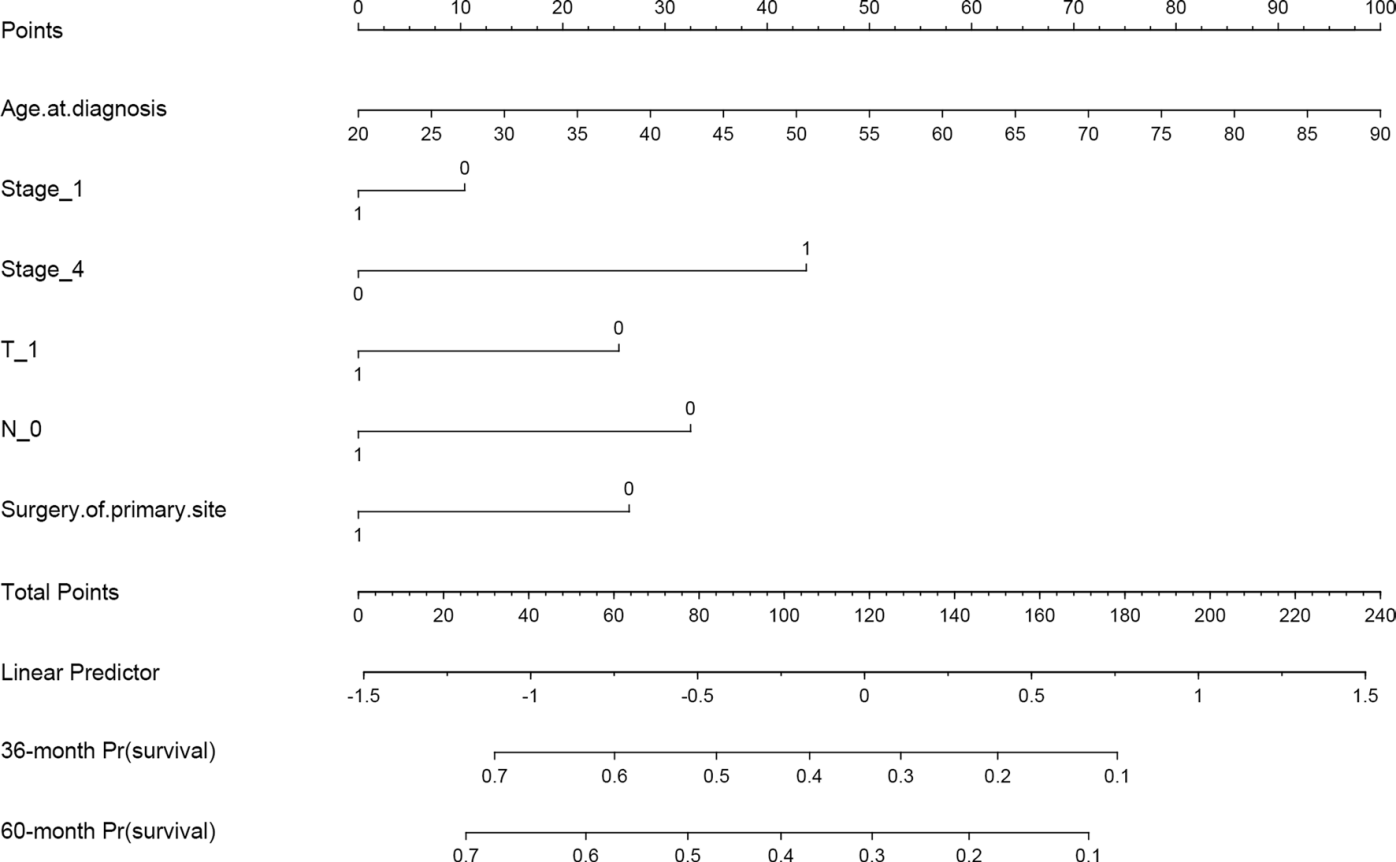
Nomogram for predicting the probability of 3- and 5-year CSS in CHGNEC patients. *CS* cancer-specific survival, *CHGNEC* Cervical high-grade neuroendocrine carcinoma.

**Fig. 3:**
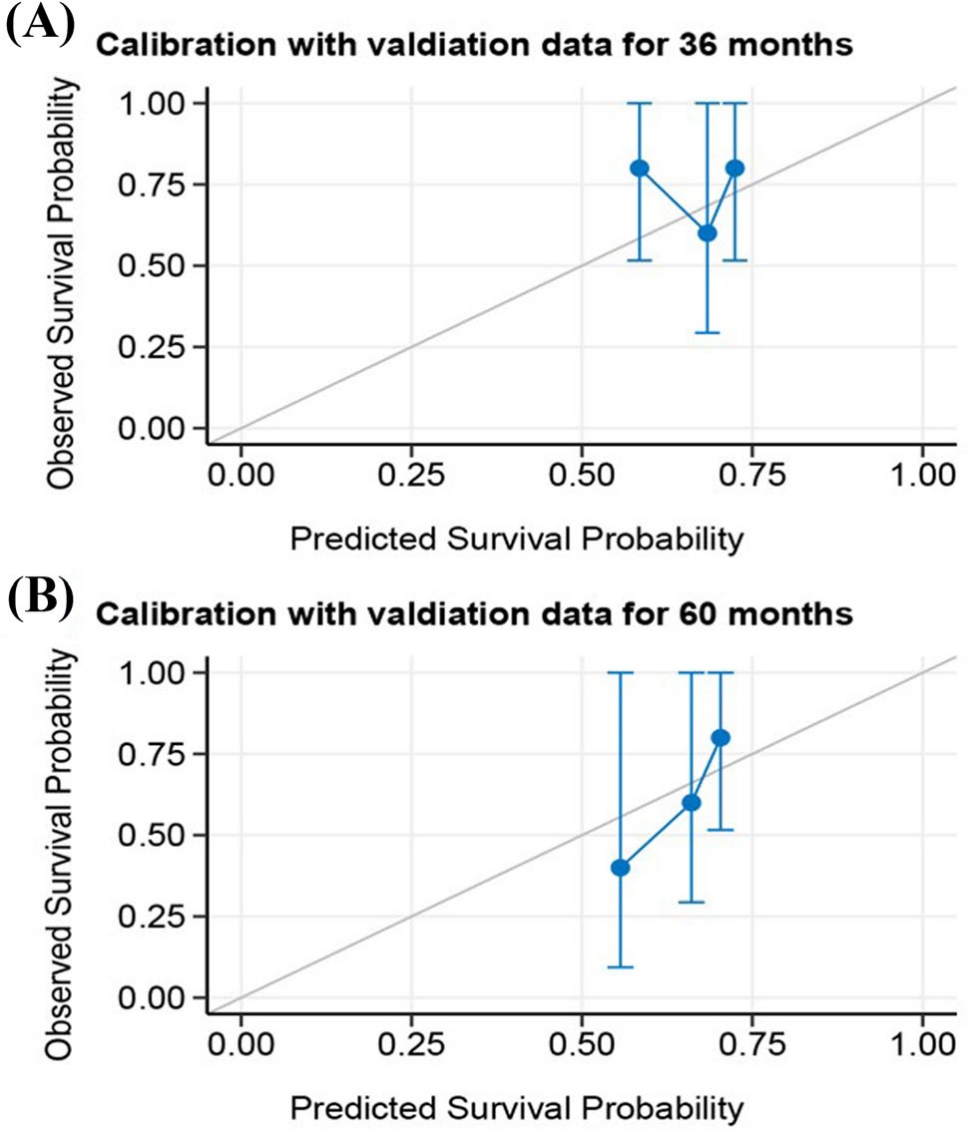
Calibration curves of the nomogram for 3- and 5-year CSS in patients. **A** 3-year and **B** 5-year calibration curves with internal validation in the development dataset; **C** 3-year and **D** 5-year calibration curves with external validation in the verification dataset. *CSS* cancer-specific survival, *CHGNEC* cervical high-grade neuroendocrine carcinoma

A Cox proportional hazards model and a RSF model were constructed using the selected predictors. To assess the models' performance, internal validation was performed using bootstrap resampling. The AUC of the Cox model at 3- and 5-year were 0.75 (95% CI 0.67–0.82) and 0.76 (95% CI 0.67–0.84), respectively. The RSF model outperformed the Cox regression, with AUCs of 0.81 (95% CI 0.75–0.87) and 0.83 (95% CI 0.77–0.89) at 3- and 5-years, respectively, as shown in Fig. 1. To aid in clinical applications, a nomogram was developed to estimate 3- and 5-years survival based on the selected parameters using the Cox regression model, as shown in Fig. 2. Internal calibration plots demonstrated good agreement between the observed and predicted rates, as shown in Fig. 3. The DCA demonstrated that both the RSF survival model and Cox model enhanced the clinical risk prediction compared to the “Reject All” or “Accept All” strategies as was shown in Supplementary Fig. 2. The net benefit from utilizing these models was evident across a threshold probability range of 20% to 80%. Notably, the RSF survival model showed greater net benefit compared to the Cox model.

### Clinical data from our institution and external validation

Over a period of ten years (March 2007–January 2017), a total of 16 patients diagnosed with CHGNEC underwent surgical intervention at our center. The median age at diagnosis was 46.5 years, and, based on the 2009 FIGO staging system, 13 cases were classified as stage I, 1 as stage II, and 2 as stage III. All patients underwent radical hysterectomy and pelvic lymphadenectomy, and postoperative chemoradiotherapy was administered to 12 patients. Additional clinicopathological characteristics of the patients are outlined in Table 3. It is noteworthy that the age (*p* = 0.343), radiation therapy (*p* = 0.106), and chemotherapy (*p* = 0.765) were similar across different datasets. However, compared to the SEER training dataset, the validation datasets displayed a tendency towards lower tumor grade and stage, as shown in Table 3.

**Table 3:** Comparison of participants’ demographic and clinical characteristics between the training and validation datasets

Characteristics	SEER (*n* = 306)	YFY (*n* = 16)	*P* value
Age at diagnosis, years			
Mean ± SD	49.9 ± 15.5	45.9 ± 10.2	
Median [IQR]	49 [37.3,60]	46.5 [39.5,51.8]	0.343
Grade			< 0.001
G2	3 (0.98%)	2 (12.5%)	
G3	118 (38.6%)	13 (81.2%)	
G4	55 (18.0%)	0 (0.00%)	
Unknown	130 (42.5%)	1 (6.25%)	
Chemotherapy			0.765
No/unknown	69 (22.5%)	4 (25.0%)	
Yes	237 (77.5%)	12 (75.0%)	
Radiation			0.106
No	119 (38.9%)	10 (62.5%)	
Yes	187 (61.1%)	6 (37.5%)	
Stage			< 0.001
I	57 (18.6%)	13 (81.2%)	
II	12 (3.92%)	1 (6.3%)	
III	75 (24.5%)	2 (12.5%)	
IV	116 (37.9%)	0 (0.00%)	
Unknown	46 (15.0%)	0 (0.00%)	
T			< 0.001
T1	95 (31.0%)	15 (93.7%)	
T2	60 (19.6%)	1 (6.3%)	
T3	73 (23.9%)	0 (0.00%)	
T4	14 (4.6%)	0 (0.00%)	
TX	64 (20.9%)	0 (0.00%)	
N			0.001
N0	104 (34.0%)	14 (87.5%)	
N1	139 (45.4%)	2 (12.5%)	
NX	63 (20.6%)	0 (0.00%)	
M			0.001
M0	149 (48.7%)	16 (100%)	
M1	112 (36.6%)	0 (0.00%)	
MX	45 (14.7%)	0 (0.00%)	
Cancer-directed surgery			< 0.001
No	191 (62.4%)	0 (0.00%)	
Yes	115 (37.6%)	16 (100%)	

Normality test for age were assessed by Shapiro–Wilk test. Comparison between groups were made using Wilcoxon rank-sum test, Chi-squared test and Fisher exact test as appropriate

*YFY* data from our hospital, *SD* standard deviation, *IQR* Inter quartile range

**Fig. 4:**
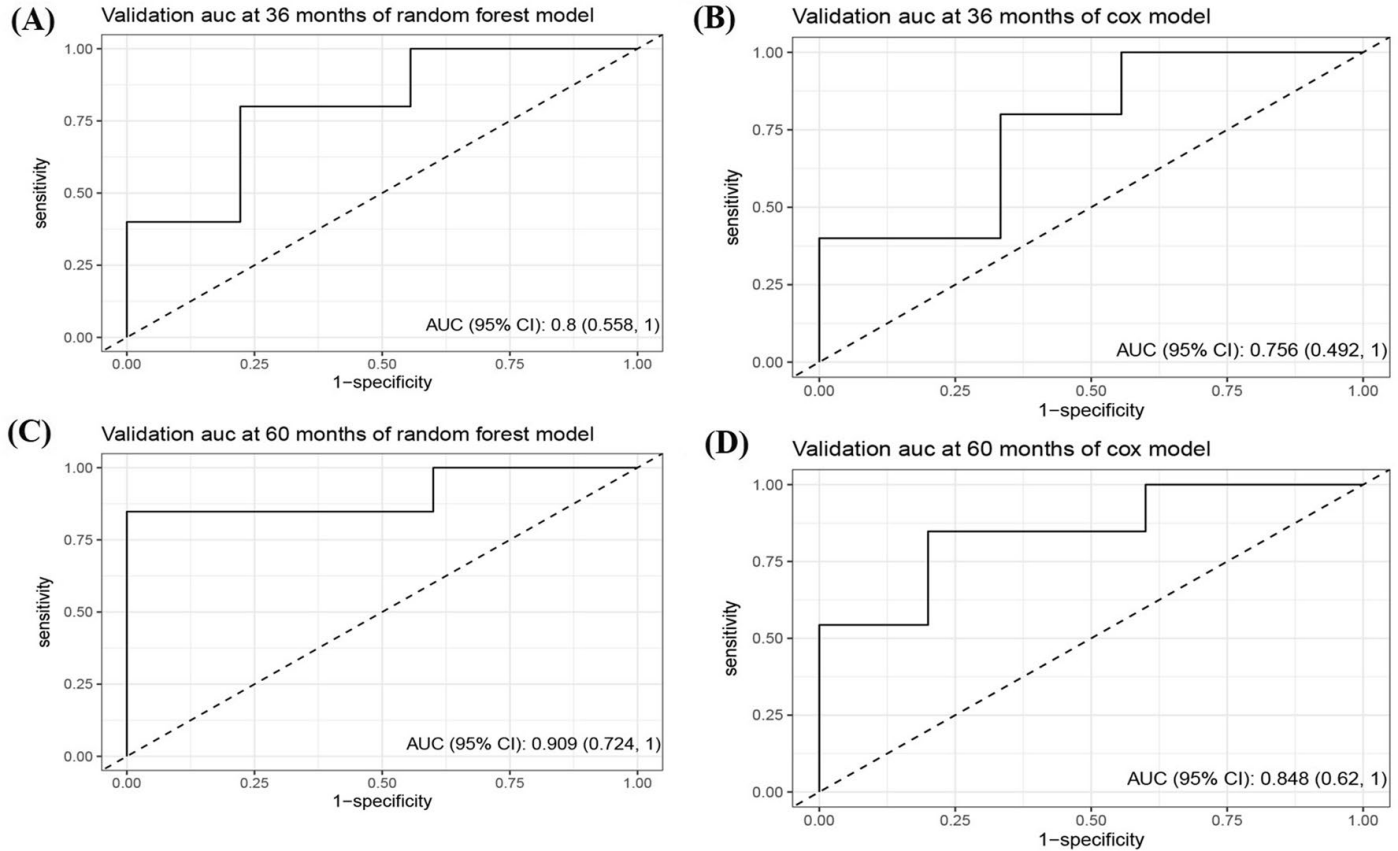
The ROC curve of predictive models for 3- and 5-year CSS in patients with CHGNEC in the external validation dataset. *ROC* receiver operating characteristic curves, *CSS* cancer-specific survival, *CHGNEC* cervical high-grade neuroendocrine carcinoma, *AUC* area under curve

In terms of external validation as shown in Fig. 4, the Cox model’s AUC for the prediction of 3- and 5-years CSS was 0.76 (95% CI 0.49–1.00) and 0.85 (95% CI 0.62–1.00), respectively. The RSF model demonstrated adequate discriminative ability in predicting CSS, with AUC values of 0.80 (95% CI 0.56–1.00) and 0.91 (95% CI 0.72–1.00) for the 3- and 5-years timepoints, respectively.

## Discussion

In the current study, we developed a prognosis prediction model using SEER database and further validated externally the model using real cases from our hospital. This study analyzed 306 patients diagnosed with CHGNEC, revealing that small cell neuroendocrine carcinoma is the most common subtype. The majority of patients were white, had insurance, and were diagnosed at stage IV. The primary treatments included chemotherapy, radiation therapy, and cancer-directed surgery. The study found that older age, advanced tumor stages, higher T stage, lymph node metastasis, and distant organ metastasis were associated with increased risk of CHGNEC-specific death, while surgery, chemotherapy, and radiation therapy were protective factors. Six variables including age at diagnosis, stage 1 status, stage 4 status, T1 status, N0 status, and surgery of the primary site were included in the final predictive model. The RSF model outperformed the Cox regression, with AUCs of 0.81 and 0.83 at 3- and 5-years, respectively. A nomogram was developed to estimate 3- and 5-years survival. The study also reported clinical data from our own institution and external validation. Overall, this study provides valuable insights into CHGNEC and highlights the importance of surgery, chemotherapy, and radiation therapy in the treatment of this disease.

Neuroendocrine tumors (NETs) are a group of rare tumors that arise from cells of the neuroendocrine system, which produces hormones and controls various physiological functions. NETs can occur in various parts of the body, including the gastrointestinal tract, lungs, pancreas, and other organs. Compared to other neuroendocrine cancers, CNEC is relatively rare. Small cell lung cancer (SCLC) is the most common subtype of neuroendocrine cancer (Meerbeeck et al. 2011), accounting for approximately 15% of all lung cancers. Gastroenteropancreatic neuroendocrine tumors (GEP-NETs) are another common subtype of neuroendocrine cancer, accounting for approximately 70% of all NETs (Cives and Strosberg 2018). These tumors arise from neuroendocrine cells in the gastrointestinal tract and pancreas. In terms of treatment, the management of neuroendocrine tumors depends on the location and extent of the tumor (Oronsky et al. 2017). Surgery is often the first-line treatment for localized tumors, followed by adjuvant therapy, such as chemotherapy or radiation therapy. For metastatic disease, systemic therapy is often used, including somatostatin analogs, targeted therapies, and immunotherapy (Mangano et al. 2016). However, the optimal treatment for CNEC is not well established, and current treatment strategies often involve a multimodal approach, including surgery, chemotherapy, and radiation therapy (Kunz et al. 2013).

Unlike squamous and adenocarcinoma subtypes, which spread primarily by local extension, cervical high-grade neuroendocrine tumors have a high rate of lymphatic and hematogenous metastasis even when disease is clinically limited to the cervix (Salvo et al. 2019). Therefore, for newly diagnosed patients, we suggest a diagnostic imaging work-up to rule out bone, liver, brain, and bone marrow metastases. The NCCN guideline for cervical cancer highly recommended a PET/CT scan for initial radiologic staging (Abu-Rustum et al. 2020).

Early prevention and screening are crucial for the effective management of high-grade neuroendocrine cervical cancer (HGNEC) due to its early hematogenous metastasis characteristic and poor prognosis. However, there is no recognized precursor for intervention prior to becoming invasive cancer. Therefore, cervical cancer prevention requires a multipronged approach involving primary, secondary, and tertiary prevention Aggarwal and (Aggarwal 2014). In terms of primary prevention, almost all HGNEC patients were infected with high-risk HPV, primarily HPV18 and HPV16. These findings are consistent with previous studies showing that most small cell neuroendocrine carcinomas (SCNC) and large cell neuroendocrine carcinomas (LCNC) are caused by HPV (Castle et al. 2018), mainly HPV18 and HPV16. HPV vaccines are effective in preventing HPV-related cancers. However, with respect to secondary prevention, cytology-based screening tests are not effective in identifying HGNEC patients. Many patients with HGNEC have normal pap smear results (Chiang et al. 2017). HPV screening strategies may be better than cytology-based screening for HGNEC, and a biopsy is recommended for patients who test positive for HPV16 and/or HPV18.

The present study has the following limitations. First, the nomogram was based on retrospective analysis, which may have caused biases due to the lack of random assignment, patient selection, and some missing values. Second, information on some potential independent prognostic variables, such as parametrial involvement, margin status, stromal invasion, and LVSI were unavailable from the SEER database, which might also increase the performance index of the model. Third, although the prediction model has been internally validated with the SEER database and externally validated using data from SFMIH, it should be further validated using data from more institutions before it is applied to the general population.

In conclusion, high-grade neuroendocrine cervical cancer is rare but vicious, more likely to suffer hematogenous metastasis and with poor prognosis. HPV test might be helpful in screening, and out nomogram is helpful in prognosis evaluation as well as personized therapy.

## Conclusions

Our study provides an excellent nomogram for predicting the prognosis of CHGNEC patients. The prognostic nomogram can be a useful tool for clinicians in identifying high-risk patients and making personalized treatment decisions.

### Supplementary Information

Below is the link to the electronic supplementary material.

**Supplementary Fig. 1** LASSO coefficient profiles of candidate predictive features The LASSO regression model was used with penalty parameter tuning that was conducted by tenfold cross-validation based on the 1 standard error of the minimum criteria (PDF 5 KB)

**Supplementary Fig. 2** Decision-curve analysis (DCA) of the clinical net benefit associated with established predictive models. The DCA demonstrated that both the RSF survival model and Cox model enhanced the clinical risk prediction compared to the “Reject All” or “Accept All” strategies (JPG 154 KB)

## Data Availability

The data presented in this study are available on request from the corresponding author. The data are not publicly available because are propriety of Shanghai First Maternity and Infant Hospital, Tongji University School of Medicine.
