# Supplementary figures and images for: Personalized prognostic prediction tool for high-grade neuroendocrine cervical cancer: a SEER database analysis and single-center validation
Source: J Cancer Res Clin Oncol. 2023 Oct 18;149(19):17395–404. doi: 10.1007/s00432-023-05414-6 (PMC10657306; doi:10.1007/s00432-023-05414-6)

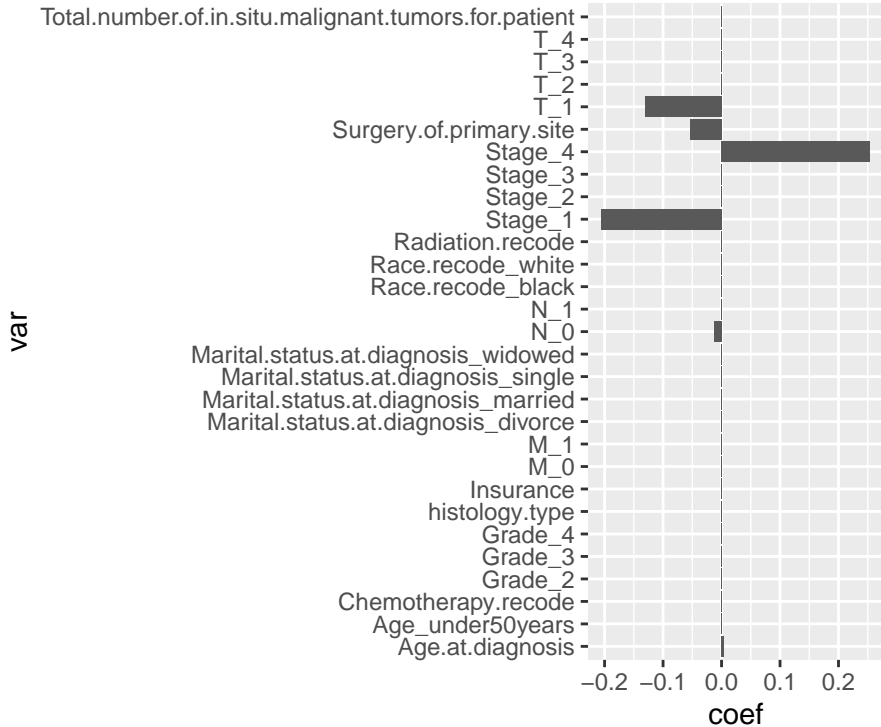

Supplement: Supplementary file 1 — Supplementary Fig. 1 LASSO coefficient profiles of candidate predictive features The LASSO regression model was used with penalty parameter tuning that was conducted by tenfold cross-validation based on the 1 standard error of the minimum criteria (PDF 5 KB) [file 432_2023_5414_MOESM1_ESM.pdf]

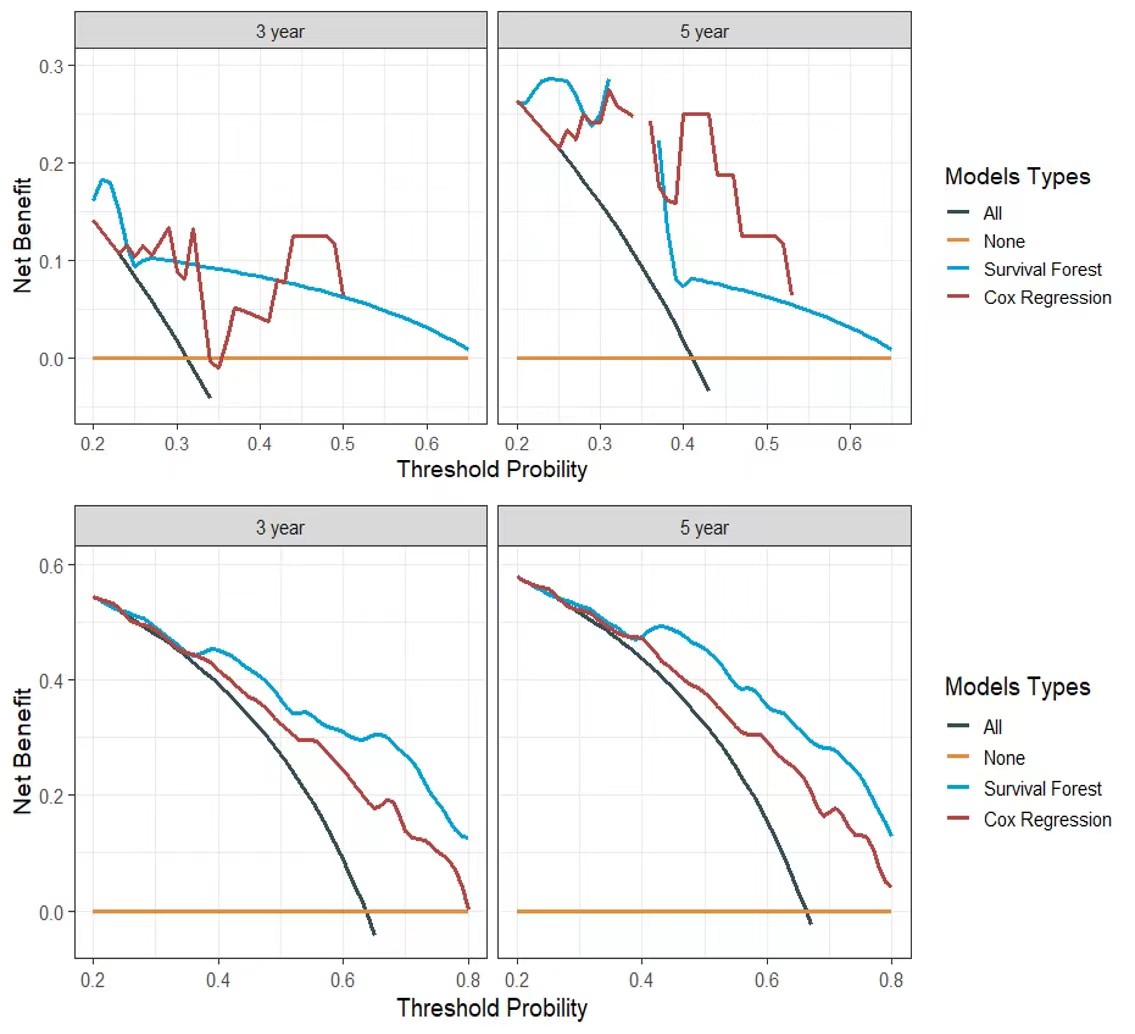

Supplement: Supplementary file 2 — Supplementary Fig. 2 Decision-curve analysis (DCA) of the clinical net benefit associated with established predictive models. The DCA demonstrated that both the RSF survival model and Cox model enhanced the clinical risk prediction compared to the “Reject All” or “Accept All” strategies (JPG 154 KB) [file 432_2023_5414_MOESM2_ESM.jpg]
